# Changes in selected hematological parameters in patients with type 1 and type 2 diabetes: a systematic review and meta-analysis

**DOI:** 10.3389/fmed.2024.1294290

**Published:** 2024-02-20

**Authors:** Getachew Mesfin Bambo, Daniel Asmelash, Ermiyas Alemayehu, Alemu Gedefie, Tadesse Duguma, Samuel Sahile Kebede

**Affiliations:** ^1^Department of Medical Laboratory Sciences, College of Health Sciences, Mizan-Tepi University, Mīzan, Ethiopia; ^2^Department of Medical Laboratory Sciences, College of Medicine and Health Sciences, Wollo University, Dessie, Ethiopia

**Keywords:** diabetic patients, hematological parameters, leukocyte count, T1DM, T2DM, red cell parameters

## Abstract

**Background:**

Diabetes mellitus is a chronic metabolic disorder that causes hyperglycemia and various life-threatening health problems. Although hematological parameters play a significant role in the progression and pathogenesis of diabetes, many studies have explored contradictory findings. Therefore, this evidence-based study aimed to determine the pooled mean difference of white blood cell and red blood cell parameters in diabetic patients in order to investigate hematological dysfunctions in type 1 and type 2 diabetes mellitus.

**Methods:**

Articles were extensively searched in bibliographic databases (PubMed, Cochrane library, Scopus, Web of Science, PsycINFO, Embase, online archives and university repositories) using appropriate entry terms. For studies meeting the eligibility criteria, the first author’s name, year of publication, study design and area, type of diabetes mellitus, sample size, and mean and standard deviation of hematological parameters were extracted using Microsoft Excel and exported to Stata 11 for meta-analysis. The pooled standardized mean difference (SMD) was determined using the random effects model, and heterogeneity was quantified using Higgins’ *I*^2^ statistics. Egger’s test and funnel plot were performed to measure bias. Furthermore, a sensitivity analysis was performed to determine the small study effect.

**Results:**

Initially 39, 222 articles were identified. After screening of the entire methodology, 22 articles with 14,041 study participants (6,146 T2DM, 416 T1DM patients and 7,479 healthy controls) were included in this study. The pooled SMD in TLC (10^9^/L) was 0.66 and −0.21, in T2DM and T1DM, respectively. Differences in absolute differential WBC counts for neutrophils, eosinophils, basophils, lymphocytes and monocytes in T2DM were 0.84, −1.59, 3.20, 0.36 and 0.26, respectively. The differences in relative differential counts (%) in T2DM were as follows: neutrophils: 1.31, eosinophils: −0.99, basophils: 0.34, lymphocytes: −0.19 and monocyte: −0.64. The SMD of differential counts of WBC (10^9^/L) parameters; neutrophils, lymphocytes, monocytes and basophils in T1DM were −0.10, −0.69, 0.19, and −0.32, respectively. The pooled SMD in RBC parameters in T2DM were as follows: RBC: −0.57 (10^6^/μL), Hb: −0.73 g/dL and HCT: −1.22%, Where as in T1DM RBC, Hb and HCT were −1.23 (10^6^/μL), −0.80 g/dL and −0.29%, respectively.

**Conclusion:**

Patients with T2DM had significantly increased TLC counts, absolute neutrophil, basophil, lymphocyte, monocyte counts and relative counts of neutrophils and basophils in comparison to controls. On the contrary, the absolute eosinophil count and relative lymphocyte, eosinophil and monocyte counts were decreased. In T1DM, WBC parameters were significantly decreased except monocytes. RBC parameters were found to be significantly decreased in T2DM patients. In T1DM, Hb and HCT were significantly decreased. However, there is no significant difference in RBC as compared with non-diabetic controls. The findings indicated a significant alteration of WBC and RBC parameters in both diabetic patients suggesting the considerable metabolic effect of diabetes on hematologic parameters.

**Systematic review registration:**

https://www.crd.york.ac.uk/prospero/export_details_pdf.php, identifier [CRD42023413486].

## Introduction

Diabetes mellitus (DM) is a chronic metabolic disorder that causes hyperglycemia and various life-threatening health problems in association with insulin secretion or action disorders. It is classified into type 1 diabetes mellitus (T1DM), type 2 diabetes mellitus (T2DM), hybrid form, gestational diabetes mellitus (GDM) and other types of diabetes (1, 2). T2DM is the most common type of diabetes, accounting for approximately 90% of all DM cases (3–6). T1DM is mainly characterized by autoimmune pancreatic B-cell destruction that leads to insulin deficiency in adults and children (7). According to current epidemiological data, around 537 million adults have diabetes mellitus and the prevalence is anticipated to rise to 783 million people globally by 2045 (3). Diabetes affects 8.8% of adults in 2015 and the proportion will increase to 10.04% in 2024 (8, 9).

Indeed, T1DM and T2DM have multiple consequences attributed to many metabolic changes including lipid metabolism, inflammation regulation, vasodilation, vascular, immunological, and hematological parameters, and cell growth (10). Chronic hyperglycemia has a higher risk of long-term damage to many vital organs, such as the eyes (retinopathy), kidneys (nephropathy), nerves (neuropathy), heart (cardiomyopathy), and blood vessels (vasculopathy), ultimately leading to a variety of diabetic complications. These complications of diabetes affect patients’ quality of life and the risk of morbidity and mortality (11, 12).

Moreover, hyperglycemia has a range of effects on RBC indices, including Hb glycation, decreased deformability, and decreased longevity (13). In diabetic patients, hematological alterations are associated with the production of reactive oxygen species (ROS) as a consequence of long-term hyperglycemia. Excessive ROS production causes oxidative stress, leading to tissue damage, hematological alterations, and endothelial and RBC dysfunction (14, 15). Patients with DM are more prone to anemia (16, 17). Several studies have revealed that total leukocyte count (TLC), Neutrophil and lymphocyte counts are higher in T2DM patients (14, 18–20).

However, some studies investigated decreased TLC and neutrophils in T2DM as compared to healthy controls (18, 21). In T1DM patients, changes in morphological and RBC and WBC counts are common in adults with T1DM (22, 23).

The majority of scientific findings demonstrated decreased WBC parameters in T1DM compared to healthy controls (22, 24, 25). In contrast, other sources reported an increase (26–28). In addition, RBC parameters are significantly decreased in T2DM (29–31). On the other hand, other studies revealed a significant increment (19, 32, 33). Cellular components of hematological parameters are altered in association with the underlying pathogenesis of DM. They are also being used to predict glycemic control and in turn various degenerative complications of DM (34). Moreover, hematological parameters play a significant role in the progression and pathogenesis of DM (35). Despite these facts, different studies have revealed inconsistent findings. The main aim of this study is to investigate the evidence-based pooled mean difference of TLC, differential WBC count, and RBC parameters in T1DM and T2DM. Therefore, the study would provide robust and sufficient evidence.

## Methods

### Design and protocol registration

This systematic review and meta-analysis were designed to estimate the mean difference in hematological parameters in diabetic patients in order to investigate hematological changes. Studies conducted on hematological parameters in T1DM and T2DM were used in this study. The result was reported in accordance with the Preferred Reporting Items for Systematic Review and Meta-analysis Protocols (PRISMA-P) (36). Moreover, the review protocol was registered in the international Prospective Register of Systematic Review (PROSPERO) under the registration number CRD42023413486.

### Eligibility criteria

#### Inclusion and exclusion criteria

(1) Community and institutional-based studies, (2) articles published in the English language, (3) studies conducted among patients with T1DM, T2DM, (4) studies published until 30 August 2023, (5) observational and experimental studies were all included. (1) Case reports, (2) abstracts without full-length articles, (3) articles with restricted access, (4) full-length articles that did not report the outcome of interest, (5) studies conducted among pregnant women, (6) patients with other complications, (7) animal studies and (8) retrospective studies were all excluded.

### Database and search strategies

Databases such as PubMed, Cochrane Library, Scopus, Web of Science and PsycINFO, Embase, online archives, and university repositories were searched. In addition grey literature including Google scholar were extensively searched. Reference lists were used to select potentially relevant studies. A comprehensive and extensive search strategy was employed using Population, Intervention, Comparator or Control and Outcome of interest (PICO) formulating questions. Appropriate entry and search terms were used by combining the “AND” and “OR” Boolean operators; ((((((((((((((Hematological profile) AND (Hematological parameters)) AND (Red blood cells)) AND (White blood cells)) AND (Biochemical profile)) AND (Blood cell indices)) AND (Diabetic patient’s)) AND (Diabetes)) AND (Non-diabetic)) AND (Type 1 diabetes)) AND (Type 2 diabetes)) AND (Glycemic control)) AND (Healthy control)) AND (Blood cell count)) OR (T1DM)) OR (T2DM). We also searched extensively for titles, abstracts, and keywords.

### Study selection and quality assessment for the risk of bias

Three authors (GB, SK, and DA) independently identified available records from repeatable databases and other sources. Initially identified records were combined into Endnot-7 to remove duplicates. Two authors (DA and SK) assessed the title, abstract and full text of the records for data abstraction. Disagreements between two independent reviewers (SK and DA) were resolved by GB for a consensus. The methodological validity of each full-length article was assessed for individual study design using the Joanna Briggs Institute (JBI) manual (37, 38). The JBI checklist of related items, sampling, eligibility protocols, description of study subject and setting, appropriate statistical analysis, case definition, confounder identification, valid and reliable result measurement, bias minimization, comparability among study participants and generalizability of the study were checked independently. The scoring system was 0 (not done), 1 (done), UC (unclear), NA (not applicable) and the judgments of the score range for cross-sectional were 0 (lowest quality) to 8 (highest quality), for case-control, 0 (lowest quality) to 10 (highest quality) for cohort 0 (lowest quality) to 11 (highest quality), experimental 0 (lowest quality) to 13 (highest quality) and quasi-experimental studies 0 (lowest quality) to 9 (highest quality) (37, 38). Articles with an average methodological score of ≥50% for each item were included in this meta-analysis (Supplementary Table S1).

### Data extraction

After careful assessment of the methodological quality of the studies, data elements were subjected to data extraction using a Microsoft Excel spreadsheet. For each article that met the eligibility protocol, the first author’s name, year of publication, study design, study area, type of DM, sample size in DM and controls, and mean and standard deviation for each hematological parameter were extracted.

### Outcome of interest

The outcome of interest was the mean difference in WBC and RBC parameters in patients with T1DM and T1DM.

### Statistical analysis

The extracted data were exported to Stata 11 for further analysis. The degree of heterogeneity was checked using Higgins’ (*I*^2^) statistics to estimate the variability in effect size estimation (4). The standard mean difference (SMD) difference was determined using a random effects model with a 95% CI (39). The pooled estimate of the SMD was presented in forest plots. In addition, publication bias was checked using a funnel plot and Egger’s statistical test. Finally, a sensitivity analysis was performed to determine the effect of a small study size on the pooled effect size. A *p*-value <0.05 was used to declare significance.

## Results

### Review process and study description

**Figure 1:**
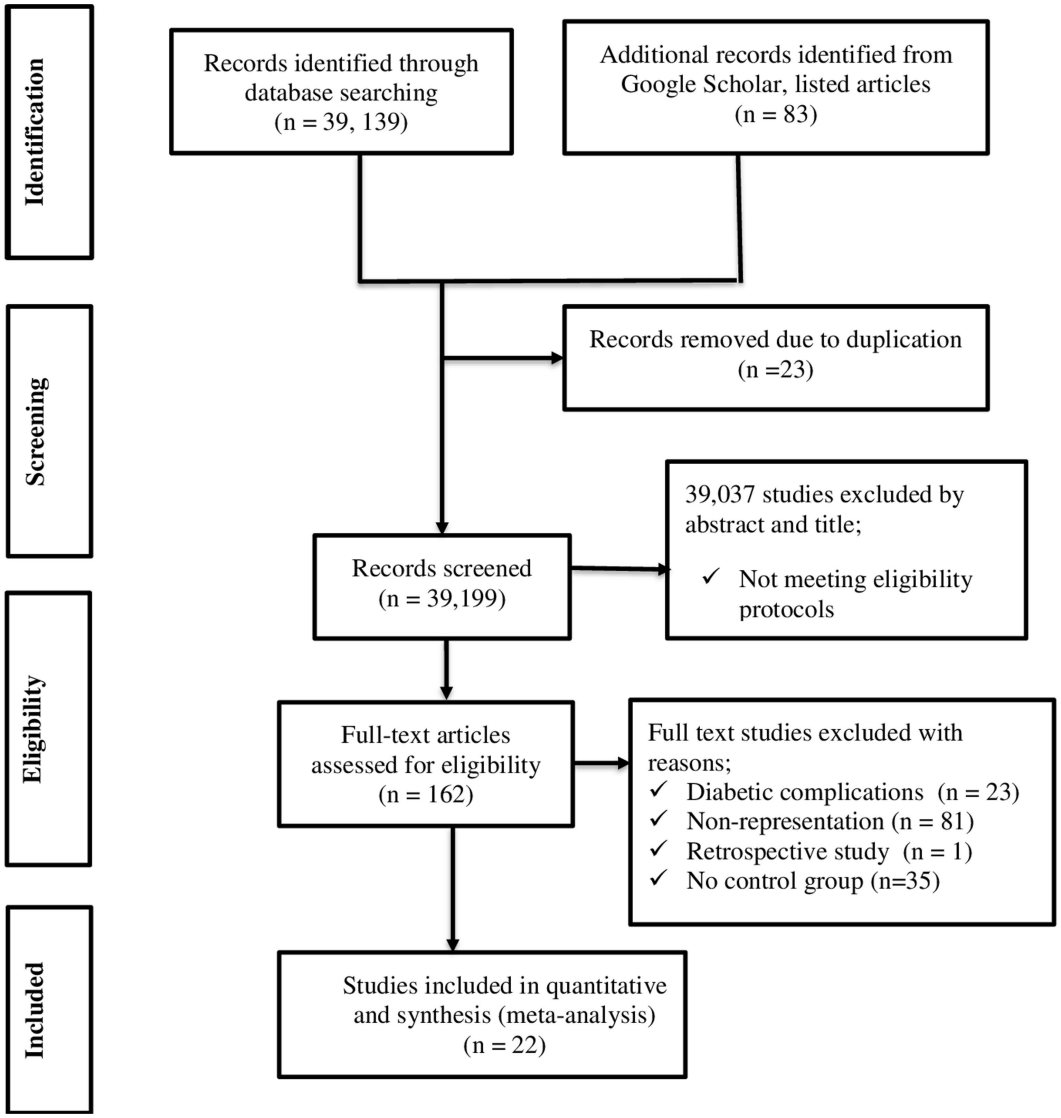
PRISMA flow chart describing screening protocols of studies for meta-analysis.

Initially, 39,222 articles were retrieved through an extensive search of electronic databases and other gray literature. From these, 23 articles were removed due to duplication. Then, approximately 39,199 records were screened for titles and abstracts. After careful assessment, 39,037 articles were discarded by abstract and title. Moreover, 162 full-length records were assessed for eligibility. Finally, 22 articles met the inclusion criteria (Figure 1).

### Characteristics of the included studies

A total of 14,041 study participants (6,146 T2DM, 416 T1DM patients and 7,479 healthy controls) were involved in the 22 included studies. Studies were conducted to assess the hematological profile in diabetic and non-diabetic patients to evaluate the changes in blood cell parameters. The majority of the studies were conducted in patients with T2DM (14, 18–21, 28–33, 40–43) and a few with T1DM (22, 24–28, 44, 45). Moreover, 19 studies were observational (14, 18–22, 25–33, 40–43, 45) and 2 were experimental studies (24, 44). One study was conducted before 2010 (24), and 21 were conducted after 2010 (14, 18–22, 25–33, 40–45) (Table 1).

**Table 1:** Summary characteristics of research articles included in quantitative analysis of systematic review and meta-analysis (*N* = 22).

Authors (references)	Year of publication	Study area	Study design	Type of DM	Sample size		Hematological profiles of the DM and control groups (Mean *±* SD)																	
							RBC parameters						Differential cell count (absolute (%) and relative count ((10^9^/L)))											
					DM	Controls	RBC-count (10^9^/L)		Hb (g/dL)		HCT %		TLC		Neutrophils		Eosinophils		Basophils		Lymphocytes		Monocytes	
							DM	Control	DM	Control	DM	Control	DM	Control	DM	Control	DM	control	DM	Control	DM	Control	DM	Control
1. Hu et al. (24)	2004	France	Quasi-experimental	T1DM	19	27	N/A	N/A	13.8 ± 3	13.5 ± 3	39.7 ± 0.8	39.3 ± 0.7	5.06 ± 0.37	5.31 ± 0.31	3.00 ± 0.33	3.14 ± 0.27	N/A	N/A	N/A	N/A	1.39 ± 0.08	1.45 ± 0.10	0.28 ± 0.02	0.26 ± 0.02
2. Umeji et al. (29)	2019	Nigeria	Case-control	T2DM	100	100	4.79 ± 0.07	5.05 ± 0.07	12.97 ± 0.19	13.8 ± 0.15	38.20 ± 0.64	42.30 ± 0.43	6.44 ± 0.25	5.66 ± 0.16 0	3.42 ± 0.21	2.51 ± 0.11	0.08 ± 0.01	0.27 ± 0.05	0.01 ± 0.00	0.04 ± 0.00	2.48 ± 0.07	2.42 ± 0.08	0.46 ± 0.04	0.46 ± 0.01
															50.81% ± 1.15	43.92% ± 1.08	1.36% ± 0.12	4.02% ± 0.67	0.15% ± 0.36	0.04 ± 0.00	40.23% ± 1.03	43.11% ± 0.90	7.08% ± 0.55	8.30 ± 0.21
3. Ebrahim et al. (21)	2022	Ethiopia	Cross-sectional	T2DM	120	120	4.89 ± 0.90	5.31 ± 0.44	13.65 ± 2.37	15.3 ± 1.49	41.08 ± 7.36	46.31 ± 4.60	6.84 ± 2.5	6.46 ± 1.60	3.96 ± 2.27	3.67 ± 1.34	0.19 ± 0.24	0.15 ± 0.10	0.04 ± 0.03	0.02 ± 0.02	2.14 ± 0.67	2.22 ± 0.51	0.51 ± 0.18	0.40 ± 0.20
															55.07% ± 13.04	55.08% ± 10.21	2.74 ± 2.72	2.44 ± 1.70	0.55 ± 0.30	0.35 ± 0.3	33.62 ± 11.55	35.97± 10.28	7.94 ± 2.73	6.14 ± 2.43
4. Pujani et al. (40)	2018	India	Cross-sectional	T2DM	30	30	N/A	N/A	13.63 ± 0.87	13.08 ± 1.31	N/A	N/A	8.79 ± 3.5	9.53 ± 2.67	N/A	N/A	N/A	N/A	N/A	N/A	N/A	N/A	N/A	N/A
5. Harish Kumar et al. (41)	2017	India	Case-control	T2DM	70	70	4.48 ± 1.64	5.12 ± 1.15	11.17 ± 4.42	14.11 ± 3.46	33.69 ± 6.48	37.27 ± 4.53	9.33 ± 3.86	7.26 ± 2.36	55.15% ± 13.97	58% ± 3.86	N/A	N/A	N/A	N/A	N/A	34.68% ± 6.46	30.28 ± 4.17	
6. Al Salhen et al. (42)	2022	Libya	Case-control	T2DM	103	39	4.24 ± 1.69	5.37 ± 0.10	12.37 ± 4.82	15.01 ± 0.49	34.49 ± 9.68	45.57 ± 2.16	9.13 ± 3.02	6.86 ± 0.63	55.15% ± 13.97	46.29% ± 4.32	N/A	N/A	N/A	N/A	32.95% ± 10.96	26.17% ± 1.83	N/A	N/A
7. Aarushi et al. (33)	2020	India	Cross-sectional	T2DM	115	115	4.63 ± 0.911	4.29 ± 0.67	12.38 ± 2.16	12.69 ± 2.02	33.52 ± 5.41	36.16 ± 5.44	8.99 ± 2.98	8.83 ± 3.48	N/A	N/A	N/A	N/A	N/A	N/A	N/A	N/A	N/A	N/A
8. Alam et al. (43)	2015	Bangladesh	Cross-sectional	T2DM	320	403	N/A	N/A	12.76 ± 1.49	13.26 ± 1.30	N/A	N/A	9.54 ± 2.65	6.74 ± 1.94	57.56% ± 9.3	61.20% ± 7.64	3.95% ± 2.79	2.09% ± 1.55	N/A	N/A	33.60% ± 8.65	29.96% ± 7.26	4.92% ± 1.52	6.58% ± 2.34
9. Adane et al. (18)	2021	Ethiopia	Cross-section	T2DM	164	82	N/A	N/A	N/A	N/A	N/A	N/A	6.95 ± 2.23	6.15 ± 1.95	50.34% ± 13.6	49.9% ± 12.53	N/A	N/A	N/A	N/A	36.17% ± 12.35	36.19%± 11.28	N/A	N/A
															3.84 ± 3.74	4.48 ± 7.33	N/A	N/A	N/A	N/A	2.81 ± 4.67	2.02 ± 0.62	N/A	N/A
10. Biadgo et al. (19)	2016	Ethiopia	Cross-sectional	T2DM	148	148	5.12 ± 0.57	5.1 ± 0.54	15.2 ± 1.7	15.1 ± 1.5	46.7 ± 5.1	46.4 ± 4.2	6.59 ± 1.42	5.56 ± 1.38	3.57 ± 1.46	3.11 ± 1.04	N/A	N/A	N/A	N/A	2.60 ± 0.70	2.04 ± 0.63	N/A	N/A
11. Arkew et al. (30)	2022	Ethiopia	Cross-sectional	T2DM	110	110	5.00 ± 0.42	5.30 ± .0.43	15.36 ± 1.2	16.50 ± 1.10	45.24 ± 3.14	47.70 ± 3.23	N/A	N/A	N/A	N/A	N/A	N/A	N/A	N/A	N/A	N/A	N/A	N/A
12. Kothari et al. (44)	2012	India	Experimental	T1DM	28	30	3.94 ± 0.5	4.77 ± 0.6	11.45 ± 1.08	12.86 ± 1.36	40.07 ± 3.87	41.56 ± 4.97	N/A	N/A	N/A	N/A	N/A	N/A	N/A	N/A	N/A	N/A	N/A	N/A
13. Ilango et al. (20)	2019	India	Case-control	T2DM	132	132	4.730 ± 0.59	4.62± 0.64	12.85 ± 1.94	13.704 ± 1.94	N/A	N/A	7.45 ± 1.98	6.61 ± 1.35	N/A	N/A	N/A	N/A	N/A	N/A	N/A	N/A	N/A	N/A
14. Abdel-Moneim et al. (26)	2020	Egypt	Case-control	T1DM	49	30	4.2 ± 0.3	4.6 ± 0.5	11.3 ± 0.9	12.6 ± 0.8	34.6 ± 3.4	37.5 ± 3.3	N/A	N/A	3.5 ± 1.10	3.2 ± 1.4	N/A	N/A	N/A	N/A	3.0 ± 0.8	2.7 ± 1.1	0.6 ± 1.3	0.4 ± 0.9
15. Arkew et al. (14)	2021	Ethiopia	Cross-sectional	T2DM	134	134	5.10 ± 0.45	5.20 ± 0.50	15.70 ± 1.20	16.20 ± 1.30	46.12 ± 3.82	46.45 ± 4.20	7.01 ± 1.74	6.50 ± 1.34	4.14 ± 1.51	3.80 ± 0.96	0.20 ± 0.1	0.10 ± 0.1	0.10 ± 0.00	0.00 ± 0.00	2.07 ± 0.62	1.86 ± 0.54	0.60 ± 0.21	0.50 ± 0.21
16. Khudhur et al. (27)	2019	Iraq	Case control	T1DM	50	50	N/A	N/A	12.61 ± 0.75	12.89 ± 0.64	N/A	N/A	9.34 ± 1.56	8.14 ± 1.88	N/A	N/A	N/A	N/A	N/A	N/A	N/A	N/A	N/A	N/A
17. Baghersalimi et al. (25)	2019	Iran	Case control	T1DM	83	83	N/A	N/A	11.4 ± 0.32	12.32 ± 0.38	N/A	N/A	6.48 ± 1.58	7.54 ± 0.71	3.63 ± 1.4	2.89 ± 0.89	N/A	N/A	N/A	N/A	2.46 ± 0.8	3.99 ± 0.54	N/A	N/A
18. Mishra et al. (45)	2013	India	Cross-sectional	T1DM	50	15	N/A	N/A	N/A	N/A	41.32 ± 3.43	43.13 ± 6.92	N/A	N/A	N/A	N/A	N/A	N/A	N/A	N/A	N/A	N/A	N/A	N/A
19. Dibby et al. (28)	2020	Iraq	Case control	T1DM	30	30	N/A	N/A	N/A	N/A	N/A	N/A	6.8 ± 1.99	5.98 ± 2.1	N/A	N/A	N/A	N/A	N/A	N/A	N/A	N/A	N/A	N/A
20. Bhatt et al. (31)	2020	India	Case control	T2DM	100	100	4.01 ± 1.52	4.82 ± 1.14	9.8 ± 2.5	12.1 ± 1.5	29.4 ± 5.28	36.3 ± 4.82	8.5 ± 3.3	7.3 ± 2.8	N/A	N/A	N/A	N/A	N/A	N/A	N/A	N/A	N/A	N/A
21. Harsunen et al. (22)	2013	Germany	Cohort	T1DM	107	1131	N/A	N/A	N/A	N/A	N/A	N/A	5.57 ± 1.37	6.89 ± 1.29	3.20 ± 1.04	4.11 ± 1.16	0.14 ± 0.10	0.15 ± 0.13	0.15 ± 0.13	0.06 ± 0.03	1.79 ± 0.54	2.11 ± 0.67	0.39 ± 0.15	0.46 ± 0.15
22. Mansoori et al. (32)	2023	Iran	Cohort	T2DM	4500	4500	4.92 ± 0.48	4.84 ± 0.48	13.81 ± 1.50	13.75 ± 1.95			6.62 ± 1.63	6.01 ± 1.53	3.63 ± 1.37	3.30 ± 3.42					2.61 ± 4.96	2.16 ± 0.89		

N/A, not available; T1DM, type 1 diabetes mellitus; T2DM, type 2 diabetes mellitus; RBC, red blood cells; Hb, hemoglobin; HCT, hematocrit; TLC, total leukocyte count; PLT, platelets; MPV, mean platelet volume; g/dL, grams per deciliter; fL, femtoliters; %, percentage of differential relative count.

### Quality and heterogeneity

The majority of the studies were of high quality, more than 75%. For individual studies, quality was assessed by using the JBI manual to minimize the risk of bias (Supplementary Table S1). The included studies exhibited substantial heterogeneity in both fixed and random effect models. To reduce the substantial heterogeneity we used the random effect model and SMD to measure the outcome of interest.

### Sensitivity analysis

When the mean difference was pooled by omitting one study at a time, each study had a negligible impact, suggesting the robustness of the pooled estimate of the mean difference (Supplementary Figures S2–S4).

### Publication bias

A funnel plot and Egger’s test were used to assess the publication bias of the included studies. Visual inspection of a funnel plot demonstrated that the articles were symmetrically distributed and fell into a funnel triangle (Supplementary Figure S1). Egger’s test showed an insignificant publication bias with a *p*-value = 0.06 (Table 2).

**Table 2:** Egger’s test.

Std. Eff.	Coef.	Std. err.	*T*	*p* > *t*	(95% conf. interval)
Slope	−0.004	0.01	0.27	0.79	−0.031, 0.04
Bias	−1.10	0.99	−2.02	0.06	−4.09, 0.09

Std. Eff., standard effect; Coef., coefficient *t*-test statistic; Std. err, standard error; *p*, *p*-value of significance by assuming a zero value; Conf. interval, confidence interval.

### Pooled estimated mean difference of hematological parameters in diabetic patients

#### White blood cell parameters in T2DM

**Figure 2:**
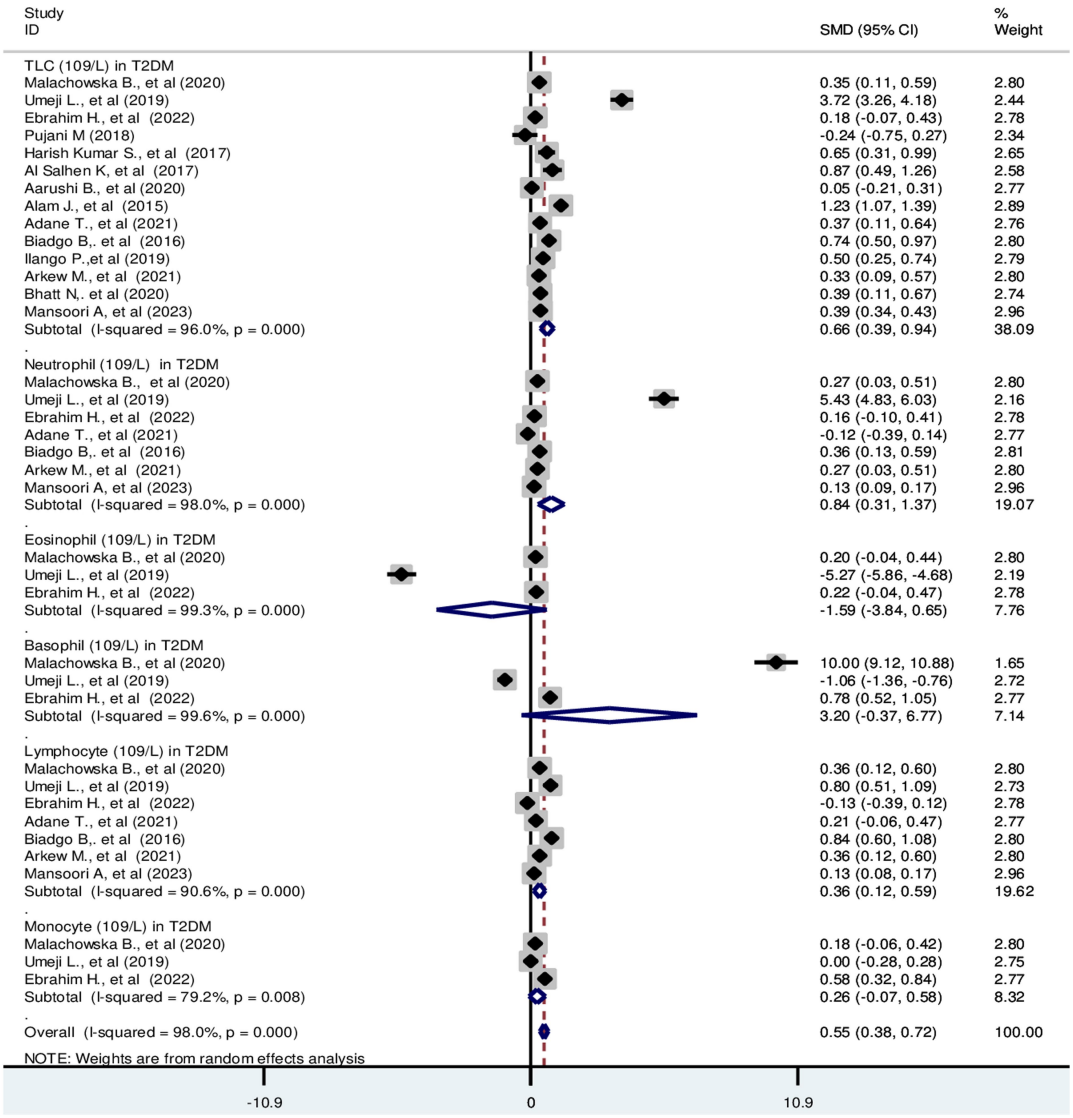
Forest plot depicting total leukocyte and absolute differential count of WBC in T2DM. The black dot in the middle of the gray box reflects the estimated mean difference of each studies point and the line shows the 95% CI of the estimates. The gray boxes represent each study weight that contributes to the estimates. I-squared illustrates the heterogeneity between the included studies. *p*-value indicates statistical significance of heterogeneity as well as mean difference.

**Figure 3:**
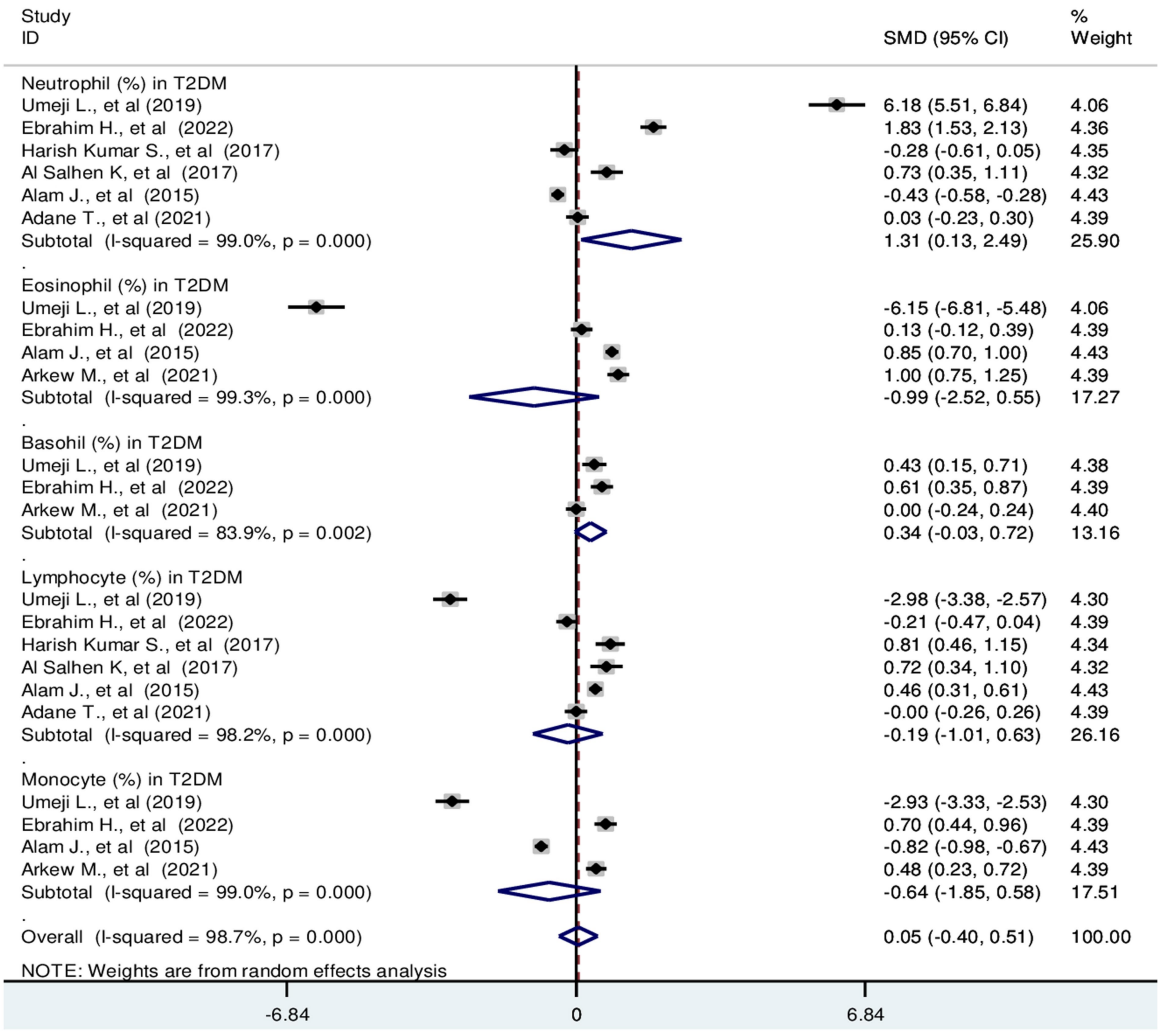
Forest plot depicting relative differential WBC count in T2DM. The black dot in the middle of the gray box reflects the estimated mean difference in relative WBC parameters of each studies point and the line shows the 95% CI of the estimates. The gray boxes represent each study weight that contributes to the contributes to the estimates. I-squared illustrates the heterogeneity between the included studies. *p*-value indicates statistical significance of heterogeneity as well as mean difference.

Based on the random effect model analysis, the pooled SMD of TLC was 0.66 (10^9^/L); 95% CI: 0.39, 0.94. Pooled SMD in neutrophils, eosinophils, basophils, lymphocytes, and monocytes were 0.84 (10^9^/L); 95% CI: 0.31, 1.37, −1.59 (10^9^/L); 95% CI: −3.84, 0.66, 3.2 (10^9^/L); 95% CI: −0.37, 6.77, 0.36 (10^9^/L); 95% CI: 0.12, 0.59 and 0.26 (10^9^/L); 95% CI: −0.07, 0.58, respectively at *p* < 0.001 (Figure 2). SMD in relative differential counts were as following; neutrophil: 1.31%; 95% CI: 0.13, 2.49, eosinophil: −0.99%; 95% CI: −2.52, 0.55, basophil: 0.34%; 95% CI: −0.03, 0.72, lymphocyte: −0.19%; 95% CI: −1.01, 0.63 and monocyte: −0.64%; 95% CI: −1.85, 0.58 at *p* < 0.001 (Figure 3).

#### White blood cell parameters in T1DM

**Figure 4:**
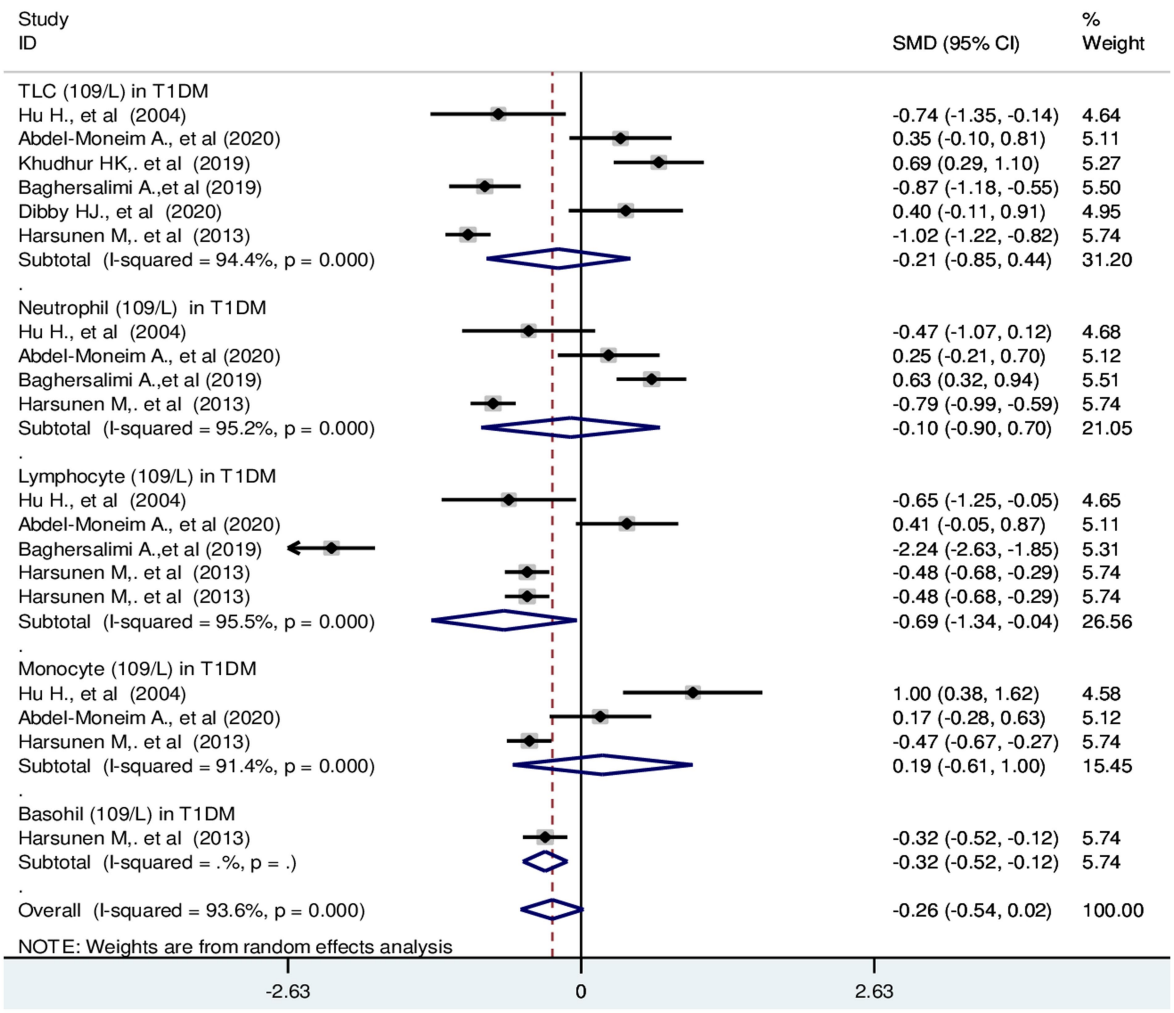
Forest plot depicting total leukocyte and differential count of WBC in T1DM. The black dot in the middle of the gray box reflects the estimated mean difference of each studies point and the line shows the 95% CI of the estimates. The gray boxes represent each study weight that contributes to the estimates.I-squared illustrates the heterogeneity between the included studies. *p*-value indicates statistical significance of heterogeneity as well as mean difference.

The pooled SMD of TLC in T1DM patients was −0.21 (10^9^/L); 95% CI: −0.85, 0.44. The SMD of differential counts of WBC parameters; neutrophils, lymphocytes, monocytes and basophils was −0.10 (10^9^/L); 95% CI: −0.90, 0.70, −0.69 (10^9^/L); 95% CI: −1.34, −0.04, 0.19 (10^9^/L); 95% CI: −0.61, 1.00 and −0.32(10^9^/L); 95% CI: −0.52, −0.12, respectively with a *p*-value <0.001 (Figure 4).

#### Red blood cell parameters In T2DM

**Figure 5:**
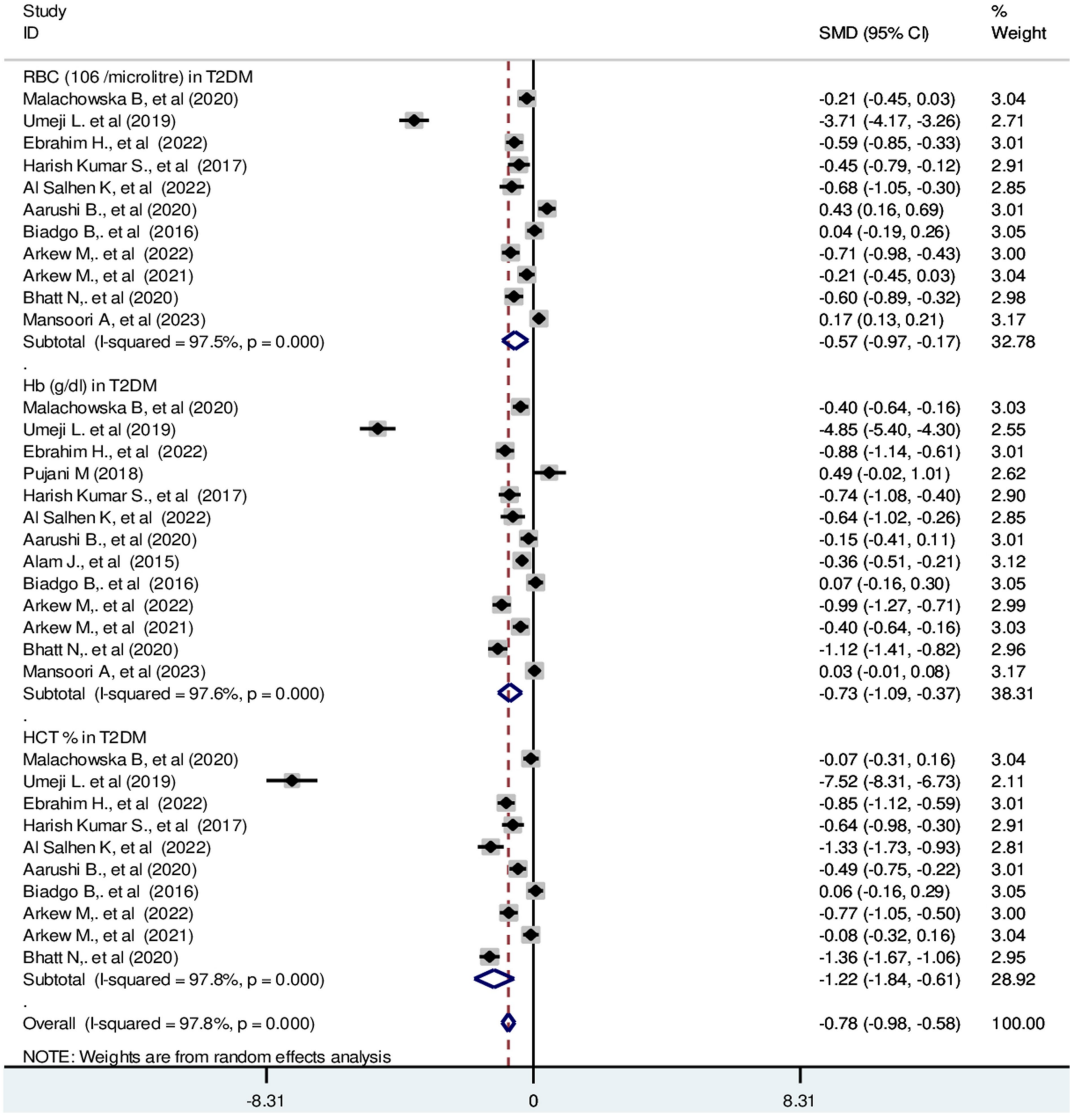
Forest plot showing RBC parameters in T2DM. The black dot in the middle of the gray box reflects the estimated mean difference of each studies point and the line shows the 95% CI of the estimates. The gray boxes represent each study weight that contributes to the estimates. I-squared illustrates the heterogeneity between the included studies. *p*-value indicates statistical significance of heterogeneity as well as mean difference.

The pooled SMD in RBC parameters in T2DM were as follows: RBC: −0.57 (10^6^/μL); 95% CI: −0.97, −0.17, Hb: −0.73 g/dL; 95% CI: −1.09, −0.37 and HCT: −1.22%; 95% CI: −1.84, −0.61 with a *p*-value <0.001 (Figure 5).

#### Red blood cell parameters in T1DM

**Figure 6:**
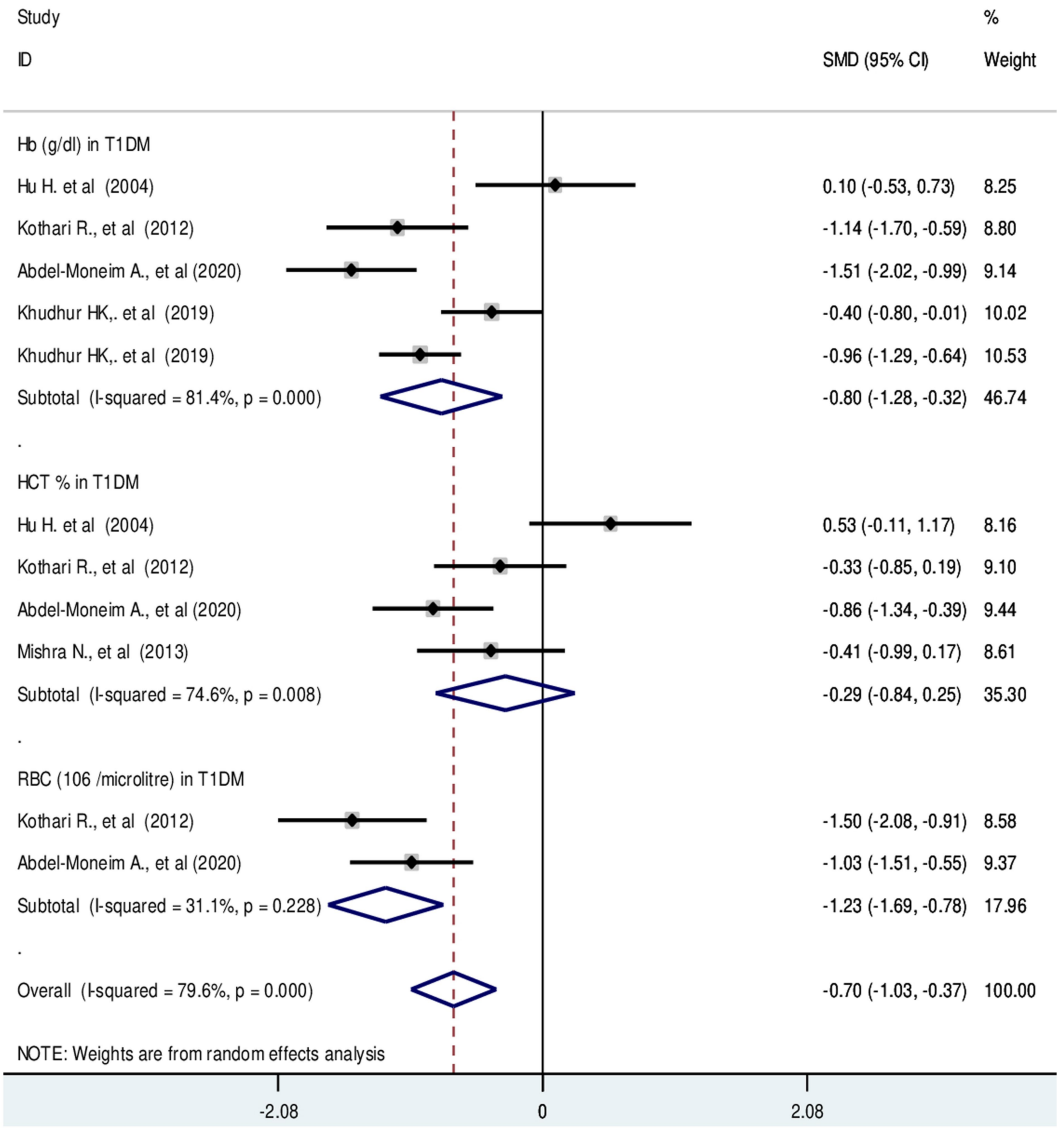
Forest plot showing RBC parameters in T1DM. The black dot in the middle of the gray box reflects the estimated mean difference of each studies point and the line shows the 95% CI of the estimates. The gray boxes represent each study weight that contributes to the estimates. I-squared illustrates the heterogeneity between the included studies. *p*-value indicates statistical significance of heterogeneity as well as mean difference.

The pooled SMD in RBC parameters in T1DM were as follows: RBC: −1.23 (10^6^/μL); 95% CI: −1.69, −0.78, *p*-value = 0.23, Hb: −0.80 g/dL; 95% CI: −1.28, −0.32, with a *p*-value <0.001 and HCT: −0.29%; 95% CI: −0.84, −0.25 with a *p*-value = 0.008, respectively (Figure 6).

## Discussion

Diabetes is a serious and chronic disease that is a major public health concern. Uncontrolled diabetes increases the risk of metabolic, cellular, and blood anomalies, which in turn lead to vascular problems, cancer, and mortality (12, 46). However, the accumulated evidence on the hematological profile of diabetic patients is less clear and contradictory for the management of patients. The main aim of this evidence-based study was to assess changes in hematological parameters (WBC and RBC) in T1DM and T2DM in order to provide accurate and substantial information for proper management of diabetes.

In this study we found that the TLC count was significantly increased in T2DM patients. Additionally, absolute differential counts of neutrophils, basophils, lymphocytes and monocytes were increased. This finding is supported by several studies (14, 18–20). However, the same finding is not supported by studies conducted in Ethiopia and India (18, 21). The possible explanation for the leukocytosis may be related to insulin resistance. Diabetes mellitus causes endothelial dysfunction, hypertension and damage to the vascular bed through a variety of biological mechanisms, including the formation of ROS, resulting in an imbalance between vasodilators and vasoconstrictors. This condition of vascular remodeling may contribute to disturbances in WBC parameters (47). Ischemic heart disease and peripheral arterial disease have also been associated with higher neutrophil counts (48, 49). These conditions are common complications of diabetes (50).

In addition, neutrophils have also been suggested as a marker of inflammation. It is a fact that chronic low-grade inflammation is involved in the pathogenesis of obese T2DM. Adipose tissue is the main trigger of low-grade inflammation in obesity-related T2DM with prominent infiltration of WBC components and immune cells (51–53).

Moreover, absolute and relative eosinophil counts decreased in T2DM as compared to healthy controls. This may be related to the systemic inflammation of diabetes. Meanwhile, eosinopenia is an emerging marker of inflammation (54). It is a fact that the release of inflammatory mediators is enhanced by hyperglycemia and diabetes-induced ROS (55). In addition, cytokines, particularly interleukin-3, interleukin-5 and granulocyte-macrophage colony-stimulating factors control and regulate the formation of eosinophils. The formation and maturation of eosinophils may be impeded by a decrease in these crucial cytokines (56). Indeed, the relative differential count of monocytes and lymphocytes was significantly decreased in T2DM.

In T1DM TLC, neutrophils, basophils and lymphocytes were found to be decreased. This finding is supported by a number of studies (22, 24, 25). A possible explanation would be cell-specific autoantibodies (22). Reduced numbers of neutrophils could be due to abnormal neutrophil yield and maturation, peripheral consumption or damage, and tissue retention (57, 58). Alteration of neutrophil migration may be another reason. The rate of neutrophil migration is lower in T1DM than in T2DM and healthy controls (59). Moreover, patients with T1DM are at risk of developing neutropenia due to neutrophil sequestration in pancreatic tissue or neutrophil infiltration of the islets of Langerhans (60–62). The neutrophil count mediates a decrease in the total WBC count. However, the finding is not supported by reports of recent studies (26–28). Surprisingly, lymphocytes reflect a calm and controlled immune response to reduce cardiac damage and decreased lymphocytes suggest an increment in apoptosis (63). Generally, perturbation of leukocyte homeostasis may indicate the involvement of the innate immune system in the progression of T1DM. Low neutrophil counts are linked with defective extravasation, a compromised bone marrow environment, a shortened half-life, increased turnover and enhanced clearance by macrophages during chronic autoimmune inflammation and islet autoimmunity (22). Additionally, T1DM is characterized by cellular-mediated autoimmune destruction of β cells in the pancreas. This aberrant T cell activation can destroy immune cells (64). Activated phagocytosis may be another explanation. Natural killer cells (NKC) can affect neutrophils during the activation of phagocytosis (65).

Moreover, this study revealed an increased monocyte count in T1DM. The possible explanation may be the severity of diabetic ketoacidosis and evidence of infection. Patients with severe diabetic ketoacidosis had higher WBC counts than those with moderate diabetic ketoacidosis (66, 67). The incidence of leukocytosis was significantly higher in patients with diabetic ketoacidosis (68). Metabolic acidosis and ketosis are the hallmarks of T1DM. In addition monocytosis may be a leukemoid reaction rather than a systemic inflammatory response. Imbalances in hormones, cytokines, and their mediators may also promote an increase in monocyte counts (67, 69).

Regarding RBC parameters; HCT, Hb and RBC mass exhibited a significant decrease in type 2 diabetic patients compared with non-diabetic controls. In T1DM, HCT and Hb values were significantly decreased. However, there is no significant difference in RBC compared with their counterparts. The possible explanation for decreased RBC parameters would be bone marrow depression, hematotoxic effects of hematopoietic precursor cells. Additionally, hyperglycemia may have long-term effects that result in the production of ROS, which could lead to irreversible glycation of Hb and RBC membranes (70, 71). Indeed, marked hematological abnormalities in children with T1DM are linked with inflammation and oxidative stress (26). Besides, hypoinsulinemia would be another reason for decreased erythropoiesis. Insulin has a synergistic effect on erythropoietin hormone stimulation. Defective iron utilization and malabsorption syndrome in association with chronic inflammation and obesity may be another explanation. Because, the central regulatory protein, hepcidin increases with diabetic-induced inflammation (72). It known that, decreased serum ferritin level in T2DM (73). Persistent hyperglycemia invariably subjects RBC to several alterations that further influence hemorheology and microcirculation (74). Moreover, increased expression of chronic hyperglycemia-induced proinflammatory cytokines like interleukin 1 and 6, tumor necrosis factor, transforming growth factor and interferons may be involved in erythroid progenitor cell apoptosis. Elevated levels of these cytokines contribute significantly to insulin resistance and cause anemia. The increase in interleukin-6 in hyperglycemic individuals has an anti-erythropoietic effect that may promote the death of immature RBCs (75).

Substantial heterogeneity was found in this study. Significant heterogeneity was observed in pooling RBC in T2DM, and WBC parameters. The possible explanations would be differences in study design, target population, statistical methods, reference range, standard operating procedures, electronic cell counters and sample size.

### Strengths and limitations of this study

The articles were thoroughly searched and retrieved. The study was conducted with high-quality records in accordance with the PRISMA guidelines. However, there were some limitations. First, the source of substantial heterogeneity was not identified. Second, only articles published in the English language were included. Third, the relative differential count of WBC in T1DM was not investigated.

## Conclusion and recommendations

Total leukocyte count and absolute differential counts; neutrophils, basophils, lymphocytes and monocytes were remarkably increased in T2DM. Additionally, the relative counts of neutrophils and basophils were increased. In contrast, the relative lymphocyte eosinophil and monocyte counts were decreased. Similarly, eosinophil count was significantly decreased in T2DM. In patients with T1DM, WBC parameters were significantly decreased except for monocytes. Hb and HCT were found to be significantly decreased in both T1DM and T2DM patients compared to healthy controls. Moreover, RBC mass was significantly decreased in T2DM, but no significant difference was found in T1DM compared with their counterparts. Comparatively, the leukocyte subset counts were lower in T1DM than in T2DM. In summary, this study demonstrated considerable changes in WBC and RBC parameters in both diabetic patients. Therefore, the findings have implications for the management of diabetic patients and highlight a diagnostic significance. Hence, early assessment and evaluation of hematological parameters is very important for the proper management of diabetes and its complications.

## Data availability statement

The original contributions presented in the study are included in the article/Supplementary material, further inquiries can be directed to the corresponding author.

## Author contributions

GB: Conceptualization, Data curation, Formal analysis, Funding acquisition, Investigation, Methodology, Project administration, Resources, Software, Supervision, Validation, Visualization, Writing – original draft, Writing – review & editing. DA: Conceptualization, Data curation, Formal analysis, Funding acquisition, Investigation, Methodology, Project administration, Resources, Software, Supervision, Validation, Visualization. EA: Conceptualization, Data curation, Formal analysis, Investigation, Methodology, Project administration, Software, Supervision, Validation, Visualization. AG: Conceptualization, Data curation, Formal analysis, Investigation, Methodology, Project administration, Software, Supervision, Validation, Visualization. TD: Conceptualization, Data curation, Formal analysis, Investigation, Methodology, Project administration, Software, Supervision, Validation, Visualization. SK: Conceptualization, Data curation, Formal analysis, Investigation, Methodology, Project administration, Software, Supervision, Validation, Visualization.

## Glossary

CI: Confidence interval
PICO: Population, intervention, comparator or control and outcome of interest
Coef: Coefficient
G/dl: Grams per deciliter
GDM: Gestational diabetes mellitus
Hb: Hemoglobin
HCT: Hematocrit
JBI: Joanna Briggs Institute
NA: Not applicable
NKC: Natural Killer cells
PRISMA-P: Preferred Reporting Items for Systematic Review and Meta-analysis Protocols
PROSPERO: Prospective Register of Systematic Review
RBC: Red blood cell
ROS: Reactive oxygen species
SMD: Standardized mean difference
Std. Eff.: Standard effect
Std. Err.: Standard error
T1DM: Type 1 diabetes mellitus
T2DM: Type 2 diabetes mellitus
TLC: Total leukocyte count
UC: Unclear
WBC: White blood cell
