# Supplementary material for: Changes in selected hematological parameters in patients with type 1 and type 2 diabetes: a systematic review and meta-analysis
Source: Front Med (Lausanne). 2024 Feb 20;11:1294290. doi: 10.3389/fmed.2024.1294290 (PMC10912516; doi:10.3389/fmed.2024.1294290)
Supplement: Supplementary file 1 [file Table_1.docx]

Supplementary table 1: The combined methodological quality of the each included studies using JBI critical appraising checklist

| **S/N** | Study ID | **Representation** | **Sampling** | **Comparability** | **Eligibility criteria** | **Data collection** | **Description of study subject** | **Valid outcome measurement** | **Bias minimization** | **Overall quality** |
| --- | --- | --- | --- | --- | --- | --- | --- | --- | --- | --- |
| 1 | Hu H., et al ([24](#_bookmark183)) | 1 | 1 | 1 | 1 | 1 | 1 | 1 | UC | High |
| 2 | Umeji L., et al ([29](#_bookmark188)) | 1 | 1 | 1 | 1 | 1 | 1 | 0 | 1 | High |
| 3 | Ebrahim H., et al ([21](#_bookmark179)) | 0 | 1 | 1 | 1 | 1 | 1 | 1 | UC | High |
| 4 | Pujani M., et al ([40](#_bookmark199)) | 1 | 1 | 1 | 1 | 1 | 1 | 1 | 1 | High |
| 5 | Harish Kumar S ., et al ([41](#_bookmark200)) | 1 | 1 | 1 | 1 | 1 | 1 | 1 | 1 | High |
| 6 | Al Salhen K, et al ([42](#_bookmark201)) | 1 | 1 | 1 | 1 | 1 | 1 | 1 | UC | High |
| 7 | Aarushi B., et al ([33](#_bookmark192)) | UC | 1 | 1 | 1 | 1 | 1 | 1 | UC | High |
| 8 | Alam J., et al ([43](#_bookmark202)) | 1 | 1 | 1 | 1 | 1 | 1 | 1 | 0 | High |
| 9 | Adane T., et al ([18](#_bookmark176)) | 1 | 1 | 1 | 1 | 1 | 1 | 1 | 1 | High |
| 10 | Biadgo B,. et al . ([19](#_bookmark177)) | 1 | 1 | 1 | 1 | 1 | 1 | 1 | UC | High |

Supplementary table 1: Continued…………………….

| **S/N** | Study ID | **Representation** | **Sampling** | **Comparability** | **Eligibility criteria** | **Data collection** | **Description of study subject** | **Valid outcome measurement** | **Bias minimization** | **Overall quality** |
| --- | --- | --- | --- | --- | --- | --- | --- | --- | --- | --- |
| 11 | Kothari et al. ([44](#_bookmark203)) | 1 | 1 | 1 | 1 | 1 | 1 | 1 | 1 | High |
| 12 | Ilango et al. ([20](#_bookmark178)) | UC | 1 | 1 | 1 | 1 | 1 | 1 | 1 | High |
| 13 | Abdel-Moneim  et al. ([26](#_bookmark185)) | 1 | 1 | 1 | 1 | 1 | 1 | 1 | 1 | High |
| 14 | Arkew et al. (14) | 1 | 1 | 1 | 1 | 1 | 1 | 1 | 1 | High |
| 15 | Khudhur et al. ([27](#_bookmark186)) | 1 | 1 | 1 | 1 | 1 | 1 | 1 | 1 | High |
| 16 | Baghersalimi et al. ([25](#_bookmark184)) | UC | 1 | 1 | 1 | 1 | 1 | 1 | UC | High |
| 17 | Mishra et al. ([45](#_bookmark204)) | 1 | 1 | 1 | 1 | 1 | 1 | 1 | 0 | High |
| 18 | Dibby et al. ([28](#_bookmark187)) | 1 | 1 | 1 | 1 | 1 | 1 | 1 | 1 | High |
| 19 | Bhatt et al. ([31](#_bookmark190)) | 1 | 1 | 1 | 1 | 1 | 1 | 1 | 1 | High |
| 20 | Harsunen et al. ([22](#_bookmark181)) | 1 | 1 | 1 | 1 | 1 | 1 | 1 | 1 | High |
| 21 | Mansoori et al. ([32](#_bookmark191)) | 1 | 1 | 1 | 1 | 1 | 1 | 1 | 1 | High |
| 22 | Arkew et al. ([30](#_bookmark189)) | 1 | 1 | 1 | 1 | 1 | 1 | 1 | Uc | High |

**Note. High quality** (75-100 %) score, (**1**) for done, (**0**) not done; **UC**: unclear. **JBI:** Joanna Brigg Institute
