# Supplementary figures and images for: Changes in selected hematological parameters in patients with type 1 and type 2 diabetes: a systematic review and meta-analysis
Source: Front Med (Lausanne). 2024 Feb 20;11:1294290. doi: 10.3389/fmed.2024.1294290 (PMC10912516; doi:10.3389/fmed.2024.1294290)

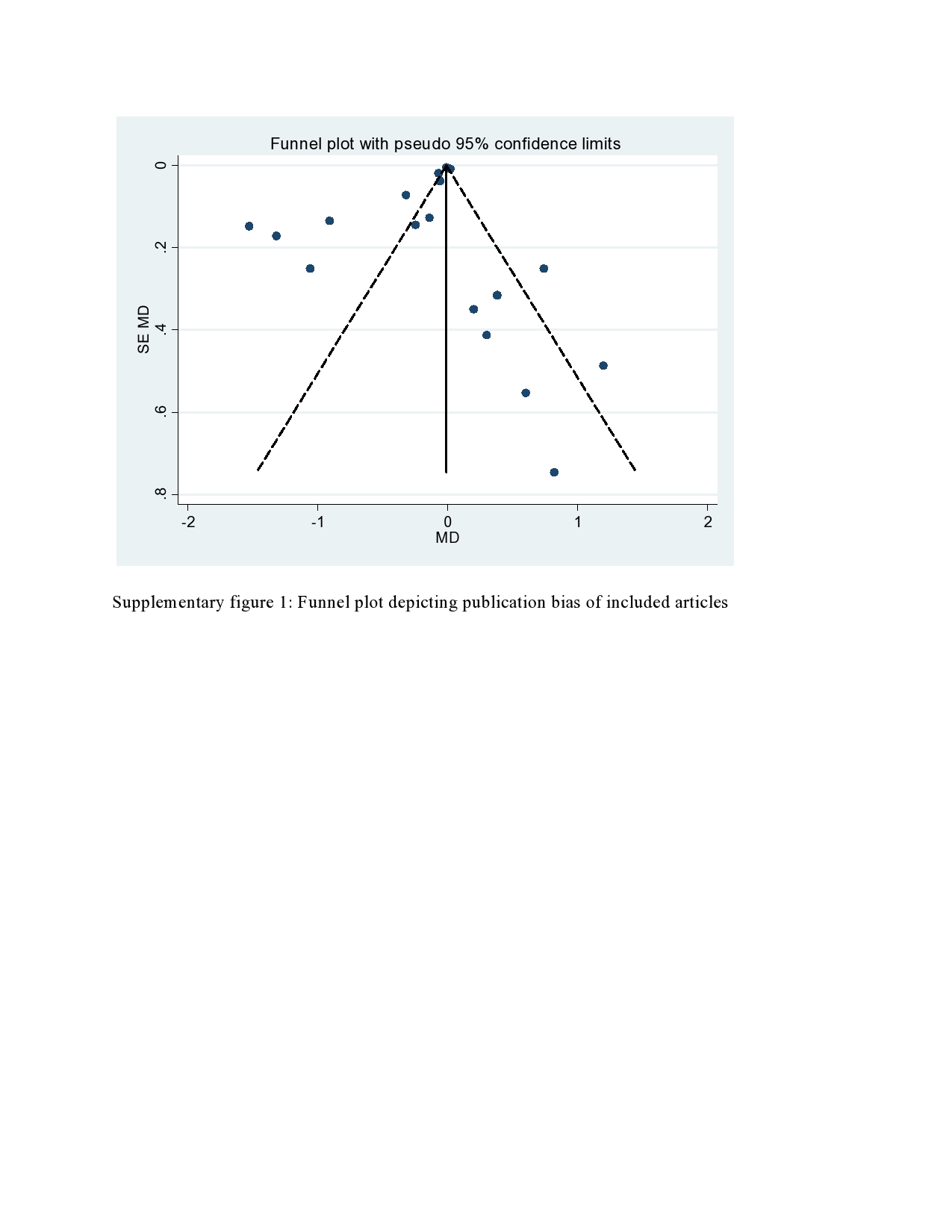

Supplement: Supplementary file 2 [file Image_1.jpg]

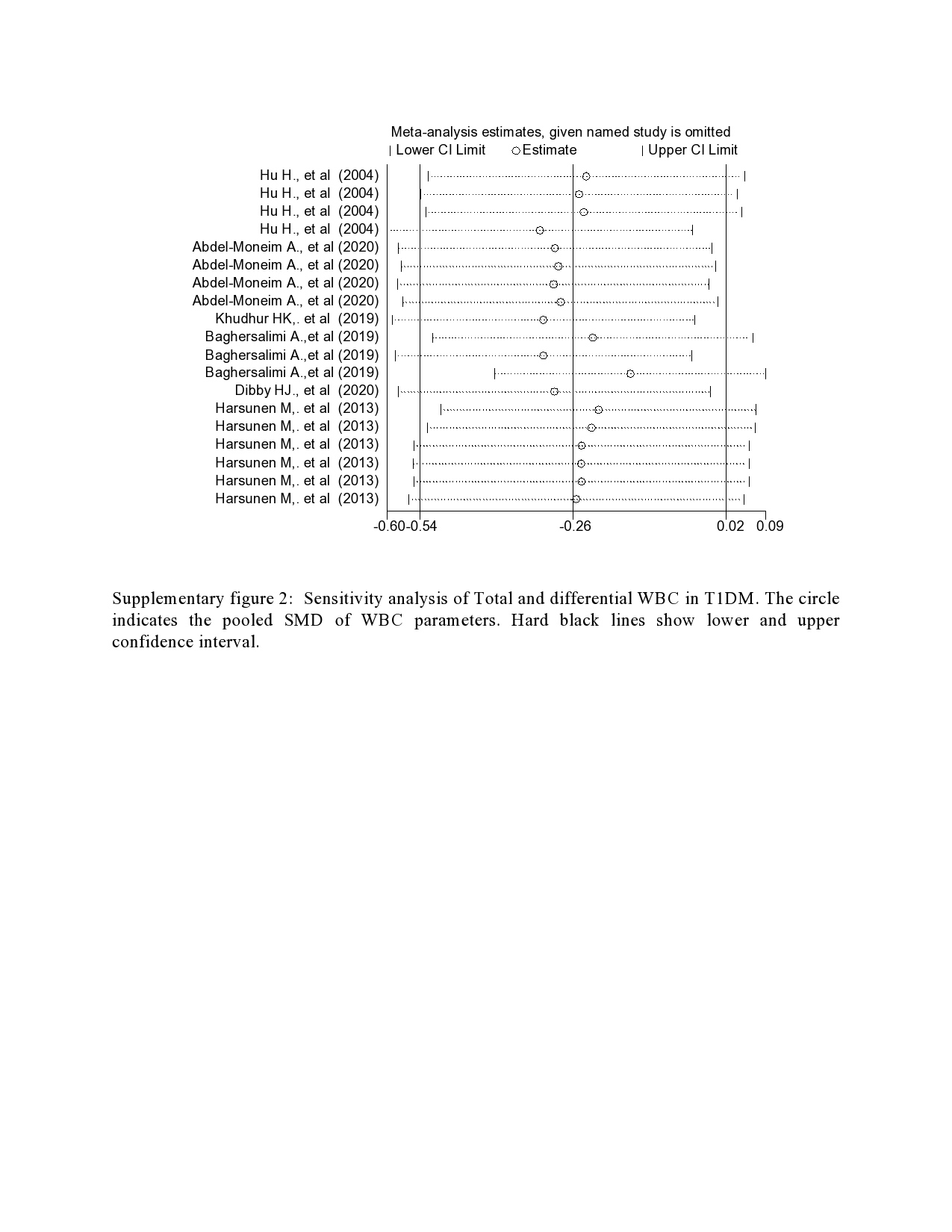

Supplement: Supplementary file 3 [file Image_2.jpg]

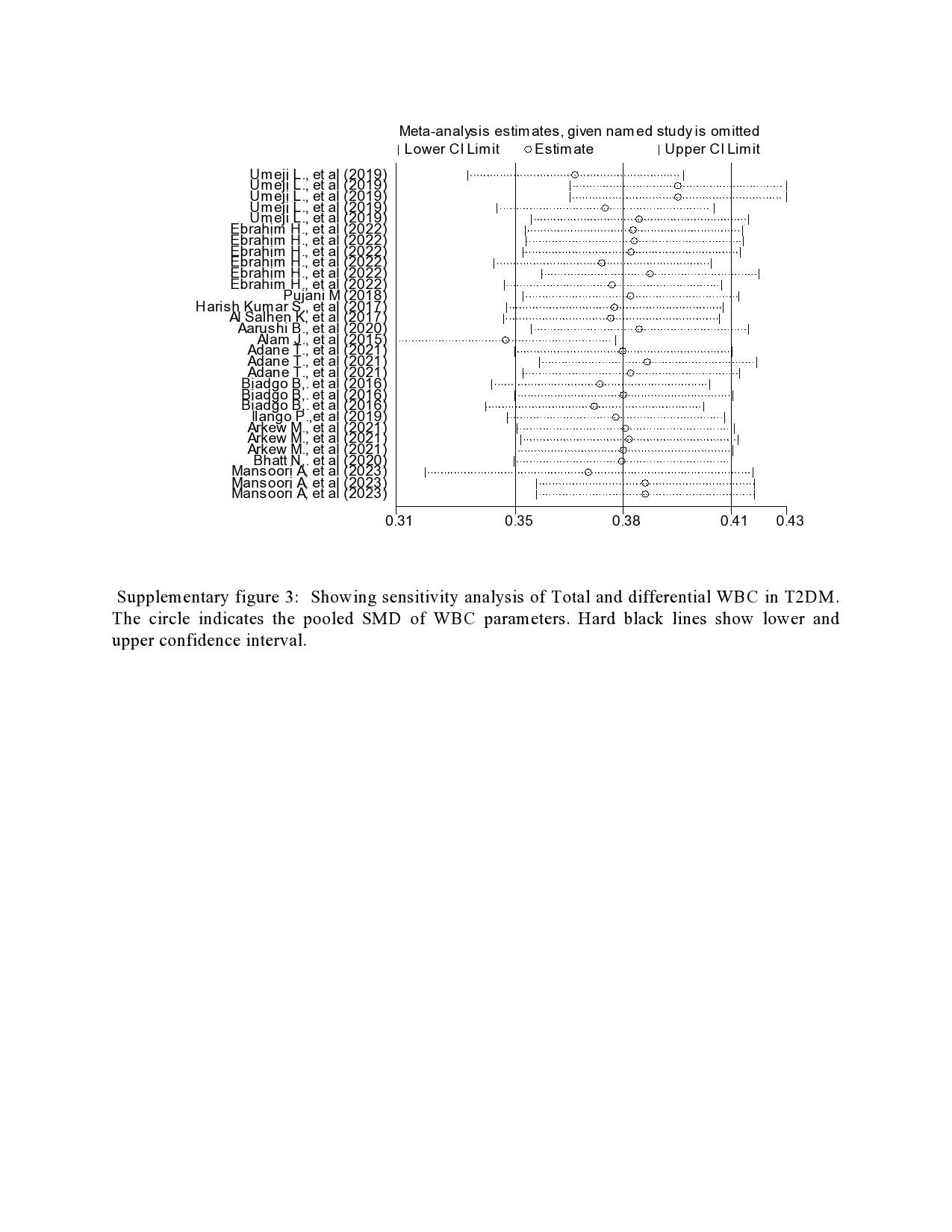

Supplement: Supplementary file 4 [file Image_3.jpg]

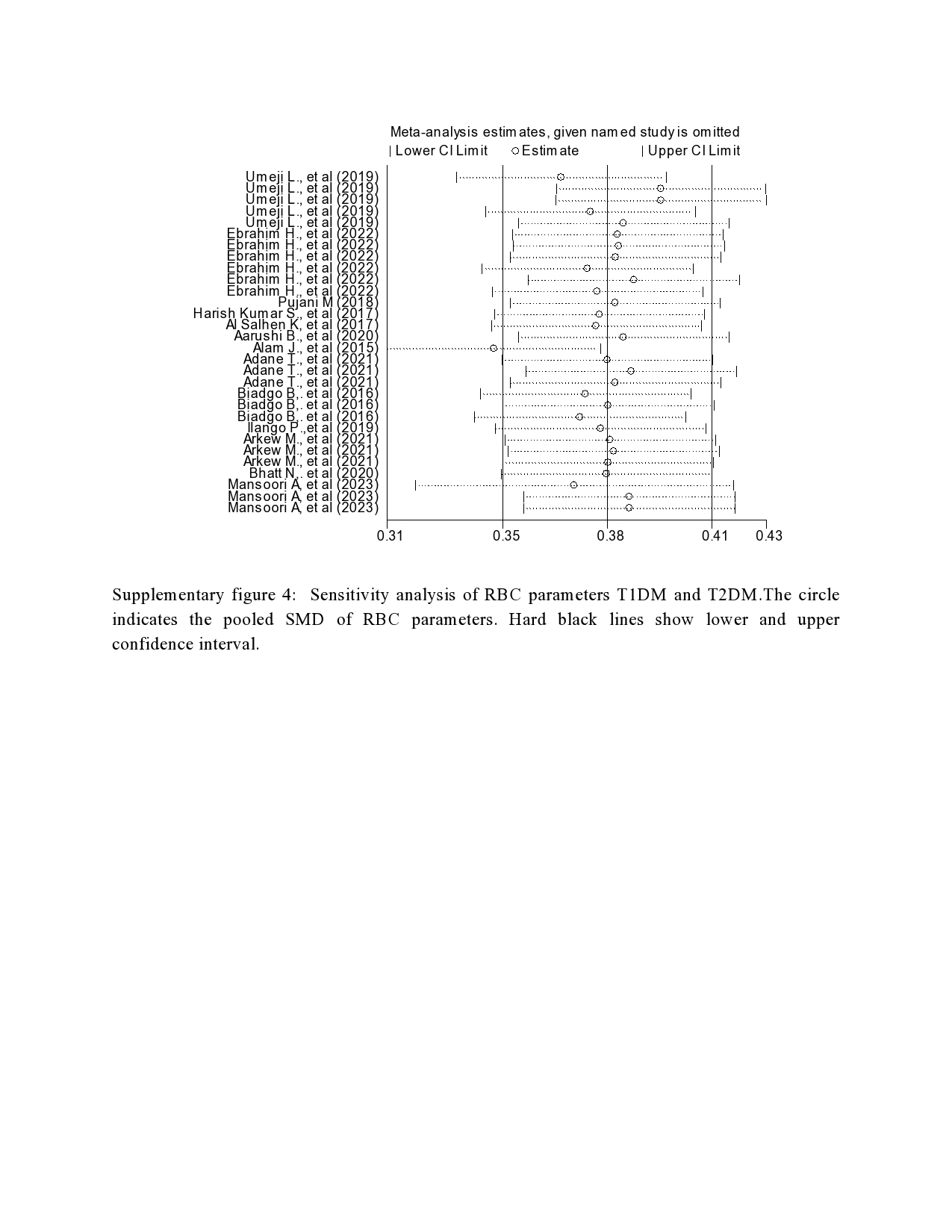

Supplement: Supplementary file 5 [file Image_4.jpg]
